# The role of spleen volume change in predicting immunotherapy response in metastatic renal cell carcinoma

**DOI:** 10.1186/s12885-023-11558-y

**Published:** 2023-10-30

**Authors:** Volkan Aslan, Atiye Cenay Karabörk Kılıç, Ahmet Özet, Aytuğ Üner, Nazan Günel, Ozan Yazıcı, Gözde Savaş, Ahmet Bayrak, Emrah Eraslan, Berna Öksüzoğlu, Hüseyin Koray Kılıç, Nuriye Özdemir

**Affiliations:** 1 Department of Medical Oncology, Gazi University, Ankara, Turkey; 2 Department of Radiology, Gazi University, Ankara, Turkey; 3 Department of Radiology, Dr. Abdurrahman Yurtaslan Ankara Oncology Training and Research Hospital, Ankara, Turkey; 4 Department of Medical Oncology, Dr. Abdurrahman Yurtaslan Ankara Oncology Training and Research Hospital, Ankara, Turkey

**Keywords:** Immune checkpoint inhibitor, Splenic volume, mRCC, Systemic inflammation

## Abstract

**Introduction:**

Resistance to immune checkpoint inhibitors (ICI) is a significant issue in metastatic renal cell carcinoma (mRCC), as it is in the majority of cancer types. An important deficiency in immunooncology today is the lack of a predictive factor to identify this patient group. Myeloid-derived suppressor cells (MDSC) are a type of cell that contributes to immunotherapy resistance by inhibiting T cell activity. While it accumulates in the tumor microenvironment and blood, it can also accumulate in lymphoid organs such as the spleen and cause splenomegaly. Therefore we aimed to evaluate the effect of increase in splenic volume, which can be considered as an indirect indicator of increased MDSC cells, on survival outcomes in mRCC patients.

**Methods:**

We analyzed 45 patients with mRCC who received nivolumab as a second-line or subsequent therapy. Splenic volume was analyzed from baseline imaging before starting nivolumab and from control imaging performed within the first 6 months of treatment initiation. Additionally, we analyzed how patients’ body mass index (BMI), IMDC risk score, ECOG performance status, nephrectomy status, neutrophil-lymphocyte ratio (NLR), Platelet-to-lymphocyte ratio (PLR) and sites of metastasis.

**Results:**

Median splenic volume change was 10% (ranging from − 22% to + 117%) during follow-up. Change in splenic volume was found to be associated with overall survival (OS) and progression-free survival (PFS) (*p* = 0.025, 0.04). The median PFS in patients with increased splenic volume was 5 months, while it was 17 months in patients without increased splenic volume. (HR 2.1, 95% CI (1–4), *p* = 0.04). The median OS in patients with increased splenic volume was 9 months, while it was 35 months in patients without increased splenic volume (HR 2.7, 95% CI (1.1–6.2), *p* = 0.025). In four patients with decreased splenic volume, neither PFS nor OS could reach the median value. Log-rank *p* value in respectively (0.015, 0.035), The group in which an increase in volume was accompanied by a high NLR had the shortest survival rate. Basal splenic volume was analyzed separately. However, neither PFS nor OS differed significantly.

**Conclusion:**

Our findings suggest that the change in splenic volume throughout immunotherapy regimens may be utilized to predict PFS and OS in mRCC patients undergoing treatment.

## Introduction

Immune checkpoint inhibitors (ICI) have revolutionized metastatic renal cell carcinoma (mRCC) treatment in recent years. Although some patients achieve strong responses, a non-negligible number of patients are resistant to ICI therapy or develop resistance within a few months. Recently, the lack of predictive factors to define this patient group is an important issue of research for immunooncology. In these studies, PD-ligand 1 (PDL1) expression was not shown to be predictive, and investigation on tumor mutation burden (TMB), tumor-infiltrating lymphocytes, and gene expression patterns is now undertaken. The IMDC risk category, on the other hand, is insufficient today since it was developed in the era of non-immunotherapy [1–3]. Therefore, the many researches are focused on to find easily accesible predictive markers for immunotherapy.

Increased Myeloid-derived suppressor cells (MDSC) have been identified as one of the important mechanisms of immunotherapy resistance in recent years. These cells constitute a diverse population of immature, immunosuppressive myeloid progenitor cells. The incidence of MDSCs increases in the spleen, the circulation of cancer patients, and the tumor microenvironment of various cancers. The increase of MDSCs in the tumor microenvironment and blood is regulated by chemokines, growth factors, and cytokines produced by the tumor [4, 5]. The increase in MDSC cells has been demonstrated through these factors, which are also increased in RCC patients [6]. MDSCs are also known as a category of cells that contribute to immunotherapy resistance by inhibiting the activity of natural killer (NK) cells and T cells and by activating immunosuppressive Tregs.

These pathological cells exert an immunosuppressive effect due to the overproduction of interleukin-10 (IL-10), transforming growth factor-β (TGFβ), arginase and nitric oxide (iNOS) in the tumor microenvironment, and also promote tumor growth by expressing T cell-inhibiting cell surface receptors [4, 7, 8]. Studies have shown that targeting and inactivating Tregs or MDSCs restores the anticancer activity of ICIs [9–11]. Previous studies in RCC patients have shown that increased MDSCs contribute to sunitinib resistance [12]. In another RCC study, the addition of MDSC-targeted therapy to IL-2 therapy was shown to increase immunotherapy response [13].

Specific genomic signatures and specific markers of MDSCs have emerged in recent years with preclinical and clinical studies, but the precise genomic signatures and surface markers that allow identification and analysis of MDSCs in clinical settings have yet to be clearly identified [8].

The spleen, which is known to influence hematopoiesis and immunological responses, is of interest for testing the efficacy of immunotherapy in numerous types of cancer. Increased MDSCs have been found to accumulate in the spleen in animal models, causing splenomegaly [14, 15]. In addition to that, in some clinical studies, MDSC level has been shown to be correlated with splenic volume [16]. Splenic volume can be quickly and easily measured in clinical practice.

Therefore, in our study, we measured splenic volume in mRCC patients before and during the first months of treatment with nivolumab. We aimed to evaluate the effect of increase in splenic volume, which can be considered as an indirect indicator of increased MDSC cells, on survival outcomes in mRCC patients.

## Materials and methods

### Study design

The patients whose splenic volume could be measured at baseline of nivolumab treatment and after three to six months of nivolumab administration were included in the study. The patient’s splenic volume was assessed both prior to beginning of the treatment with nivolumab and at the first radiolgical evaluation of the following the treatment. In between September 2010 and September 2021, at two oncology clinics, we examined 45 patients with metastatic renal cell carcinoma who received nivolumab as a second- or third-line treatment following tyrosine kinase inhibitors. The study was approved by an ethics committee, and throughout the data collection and processing, all ethical rules were obeyed.

### Study population and data

**Fig. 1:**
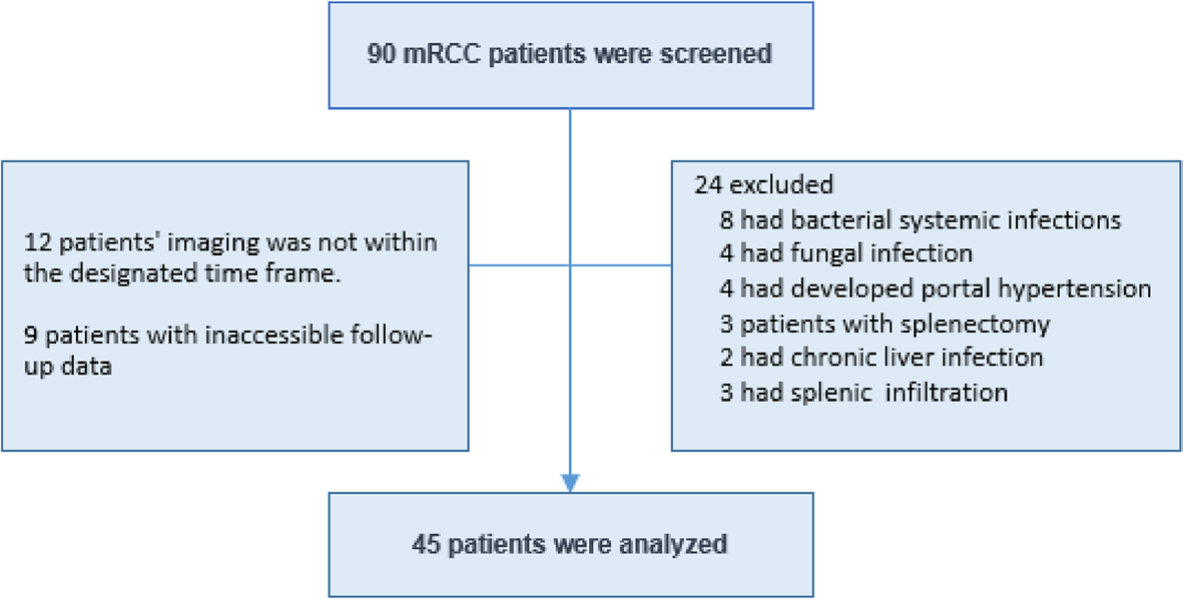
Study flowchart. Screening failures (the disease might possibly change splenic volume and Inadequacy of imaging criteria)

The patients in this trial were adults with a histologically confirmed diagnosis of mRCC. An abdominal computed tomography was performed on each patient. Within one month prior to beginning nivolumab as well as between three and six months after beginning nivolumab. Patients with various disorders were excluded from the trial such as, autoimmune diseases, chronic liver disease and systemic infections that might possibly change splenic volume. The patients with known infectious (hepatitis B or C, tuberculosis, brucellosis or candidemia) disease were excluded from study population (Fig. 1). Two weeks prior to the first nivolumab dose, clinical and laboratory data were evaluated. Absolute neutrophil and lymphocyte counts (NLR) and Platelet-to-lymphocyte ratio (PLR) were taken for each patient. The median NLR and PLR values were 3.40 and 206, respectively.

### Spleen volume estimation

The baseline spleen volume was determined through imaging conducted within the 4 weeks immediately preceding the initiation of nivolumab treatment. Subsequent volume changes were assessed through control imaging performed after the commencement of nivolumab treatment, typically following an average of 6–8 administrations of nivolumab. A twenty years experienced radiologist blinded to clinical outcomes made measurements on axial and coronal reformatted computed tomography images to calculate splenic volume. A formula derived by Prassopoulos P. et al. (ref) was used to determine splenic volume. The formula is: V = 30 + 0.58 (W × L × T), W: the maximal width of spleen at the hilum, T: the maximum thickness at hilum on a plane perpendicular to the W, L: maximal caudocranial length [17].

### Evaluation and statistical analyses

Overall survival (OS) was measured as the time from the first dose of nivolumab to death from any cause. The progression-free survival (PFS) was time from first dosage of nivolumab to disease progression or death, or date of patients last outpatient clinic visit. The radiological response was evaluated using RECIST version 1.1 criteria.

In order to conduct statistical analysis, the SPSS program, version 23, was utilized. The Kaplan-Meier (log rank) test was utilized in the calculations for the survival analyses. In order to calculate hazard ratios, In both univariate and multivariate analyses, we made use of the Cox proportional hazards model (HRs). Cox regression analysis was utilized to identify factors that could serve as independent predictors of mRCC survival rates. These were assessed individually for PFS and OS. In the interpretation of all the results, a statistical significance level of p less than 0.05 was regarded to exist.

## Results

### Patient characteristics

Our recruitment criteria were met by 45 patients, who were therefore included in this study. Table 1 shows the demographics of patients. Prior to receiving nivolumab, all patients had previously been treated with at least one line of tyrosine kinase inhibitor (TKI) treatment. The patients had been using nivolumab intravenously at a dose of maximum 240 mg every 2 weeks. No Immune-Related Adverse Events (irAEs) had occurred in any patient. The most common site of metastasis in all patients was the lungs. Radiotherapy was used as a local treatment for one patient with brain metastases.

### Increase in splenic volume following immunotherapy initiation

The median basal splenic volume of all patients was 280 mL (range: 108–769). Median splenic volume change was 10% (ranging from − 22% to + 117%) during follow-up. A change of at least 10% in splenic volume was accepted as an increase or decrease After nivolumab treatment, an increase in spleen size was observed in 22 (49%) patients, while no increase was observed in 23 (51%) patients. In four patients, the spleen volume decreased by at least 10%.

**Table 1:** Patient characteristics (n 45)

		Splenic Volume		
Group	Total n %	Not increased	İncreased	*P*
Age				
< 65 years	29 (64)	16 (69.6)	13 (59.1)	0.46
≥ 65 years	16 (36)	7 (30.4)	9 (40.9)	
Gender				
Female	14 (31)	13 (56.5)	19 (86.4)	**0.02**
Male	31 (69)	10 (43.5)	3 (13.6)	
ECOG performance status				
0–1	34 (76)	18 (78.3)	16 (72.7)	0.66
≥ 2	11 (24)	5 (21.7)	6 (27.3)	
Nephrectomy				
Yes	33 (73)	4 (17.4)	8 (86.4)	0.15
No	12 (27)	19 (82.6)	14 (13.6)	
IMDC Risk Group				
Favorable	7 (16)	3 (13)	3 (13.6)	0.49
Intermediate	36 (80)	20 (87)	17 (77.3)	
Poor	2 (4)	0 (0)	2 (9.1)	
Tumor Histology				
Clear Cell	39 (87)	19 (82.6)	20 (90.9)	0.66
Non-clear Cell	6 (13)	4 (17.4)	2 (9.1)	
Body Mass Index Status (kg/ m^2^)				
< 25	22 (49)	9 (39.1)	13 (59.1)	0.42
25-29.9	14 (31)	9 (39.1)	5 (22.7)	
≥ 30	9 (20)	5 (21.7)	4 (18.2)	
Number of prior systemic therapies				
1	21 (47)	9 (39.1)	13 (59.1)	0.18
2	24 (53)	14 (60.9)	9 (40.9)	
Sites of metastases at baseline				
Lung	24	14	10	
Liver	7	2	4	
Bone	14	8	5	
Lymph node	12	7	6	
Brain	1	0	1	
Others	4	2	2	

### Survival and prognosis

The outcomes of our univariate and multivariate OS and PFS analyses are shown in Tables 2 and 3. Change in splenic volume was found to be associated with OS in univariate analysis (*p* = 0.025). Although the NLR and PLR results were borderline insignificant (*p* = 0.072, 0.052), they were included in our multivariate analysis with splenic volume because they are clinically important parameters. In the multivariate analysis, only splenic volume significantly affected overall survival (OS) (Table 2). Change in splenic volume was found to be associated with PFS in univariate analysis (*p* value 0.04) (Table 3).

**Table 2:** Univariate and multivariate analyses for overall survival (OS)

Characteristics	n %	Survival (month) mOS	Univariate analyses HR 95% CI p		Multivariate analyses HR 95% CI p
**Age**					
< 65y	29 (64)	33	1		
≥ 65y	16 (36)	13	1.6 (0.7–3.7)	0.24	
**Gender**					
Male	31 (69)	18	1		
Female	14 (31)	25	1.4 (0.4–2.4)	0.78	
**ECOG**					
0–1	34 (76)	25	1		
≥ 2	11 (24)	8	1.7 (0.7–4.1)	0.25	
**IMDC Risk Group**					
Favorable	7 (16)	34	1		
Intermediate	36 (80)	18	1.3 (0.3–5.6)	0.70	
Poor	2 (4)	1	1.6 (0.2–13)	0.60	
**Tumor Histology**					
Non-clear Cell	6 (13)	NE	1		
Clear Cell	39 (87)	25	0.8 (0.2–3.8)	0.86	
**BMI kg/ m**^**2**^					
< 25	22 (49)	18	1		
25-29.9	14 (31)	14	1.1 (0.4–2.6 )	0.90	
≥ 30	9 (20)	24	0.7 (0.2–2.7 )	0.70	
**Basal Splenic Volume**					
< median 280 ml	23 (51)	25	1		
≥ median 280 ml	22 (49)	18	0.9 (0.4–2.2)	0.94	
**Splenic Volume (%)**					
Not increased	23 (51)	35	1.0		
İncreased	22 (49)	9	**2.7 (1.1–6.2)**	**0.025**	**2.5 (1–6) 0.048**
**NLR**					
<median 3.40	19 (42)	33	1		
≥median 3.40	26 (58)	15	2.5 (0.9–6.7)	0.072	2.4 (0.9–6.7) 0.075
**PLR**					
<median 206	20 (44)	47	1		
≥median 206	25 (56)	10	2.5 (0.9–6.5)	0.052	2.1 (0.8–5.6) 0.11

- Median survival calculated with Kaplan–Meier method

- Regression coefficient, HRs, and *P* values calculated with the Cox proportional hazards model

**Table 3:** Univariate analyses for progression-free survival (PFS)

	n %	Survival (month) mPFS	Univariate analyses HR 95% CI p	
**Age**				
< 65y	29 (64)	11	1	
≥ 65y	16 (36)	6	1.4 (0.7-3)	0.31
**Gender**				
Male	31 (69)	7	1	
Female	14 (31)	7	1.1 (0.5–2.5)	0.75
**ECOG**				
0–1	34 (76)	7	1	
≥ 2	11 (24)	6	1.1 (0.4-2)	0.95
**IMDC Risk Group**				
Favorable	7 (16)	4	1	
Intermediate	36 (80)	7	0.5 (0.3–5.6)	0.20
Poor	2 (4)	1	0.7 (0.2–13)	0.70
**Tumor Histology**				
Non-clear Cell	6 (13)	7	1	
Clear Cell	39 (87)	7	1 (0.3–3.4)	0.90
**BMI kg/ m**^**2**^				
< 25	22 (49)	7	1	
25-29.9	14 (31)	4	1 (0.5–2.6 )	0.70
≥ 30	9 (20)	5	0.9 (0.3–2.5 )	0.90
**Basal Splenic Volume**				
< median 280 ml	23 (51)	5	1	
≥ median 280 ml	22 (49)	7	0.9 (0.4–1.8)	0.72
**Splenic Volume (%)**				
Not increased	23 (51)	17	1.0	
İncreased	22 (49)	5	**2.1 (1–4)**	**0.04**
**NLR**				
<median 3.40	19 (42)	8	1	
≥median 3.40	26 (58)	5	1.7 (0.8–3.5)	0.19
**PLR**				
<median 206	20 (44)	11	1	
≥median 206	25 (56)	5	1.6 (0.8–3.5)	0.19

- Median survival calculated with Kaplan–Meier method

- Regression coefficient, HRs, and *P* values calculated with the Cox proportional hazards model

**Fig. 2:**
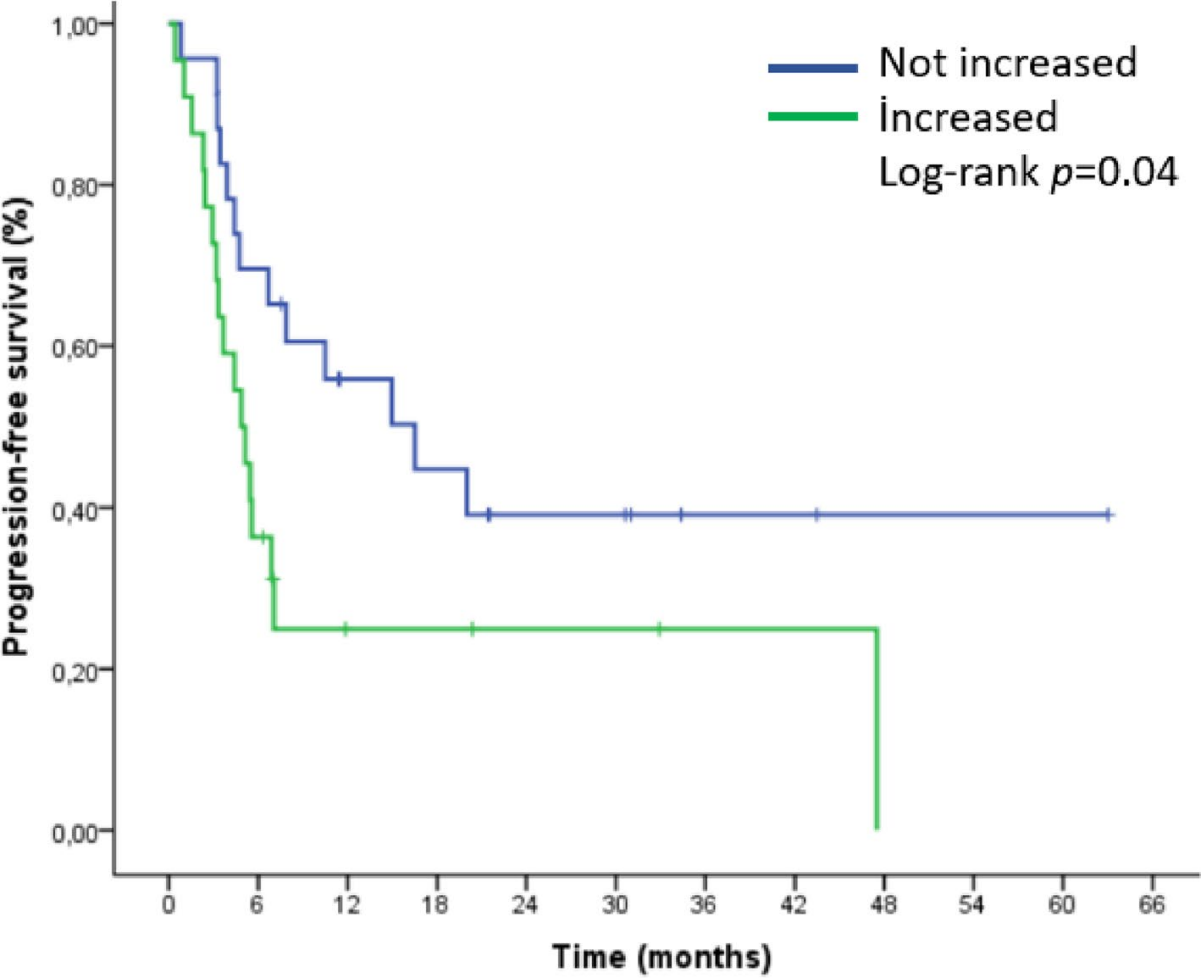
Kaplan-Meier curve for progression-free survival (PFS) based on splenic volume change after nivolumab initiation

**Fig. 3:**
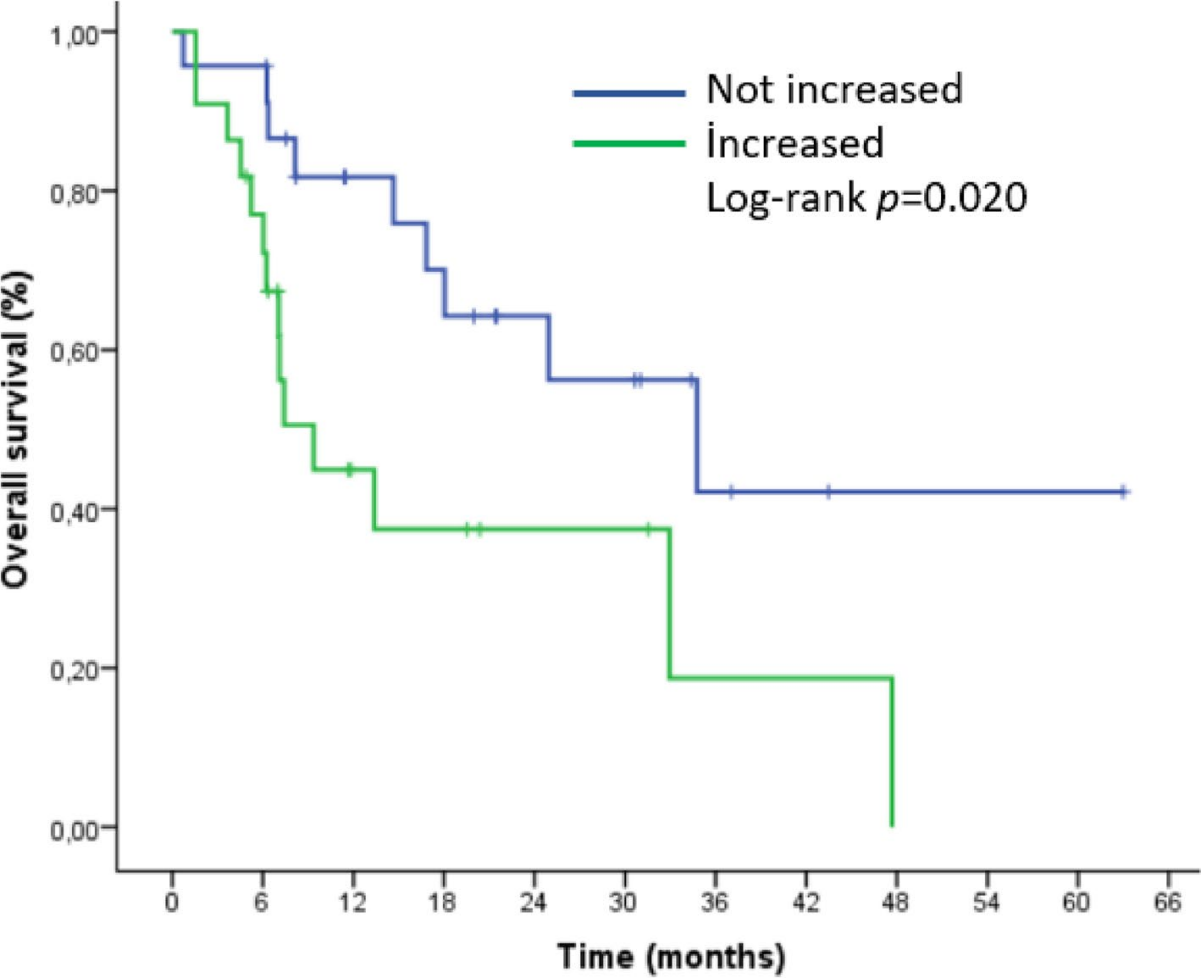
Kaplan–Meier curve for overall survival (OS) based on splenic volume change after nivolumab initiation

The median progression-free survival (mPFS) in patients with increased splenic volume was 5 months, while it was 17 months in patients without increased splenic volume. (HR 2.1, 95% CI [1–4], *p* = 0.04). Figures 2 and 3 demonstrated the outcomes of our Kaplan-Meier analysis of these two groups.

The median overall survival (mOS) in patients with increased splenic volume was 9 months, while it was 35 months in patients without increased splenic volume. (HR 2.7, 95% CI (1.1–6.2), *p* = 0.025). Figures 2 and 3 demonstrated the outcomes of our Kaplan-Meier analysis of these two groups.

**Fig. 4:**
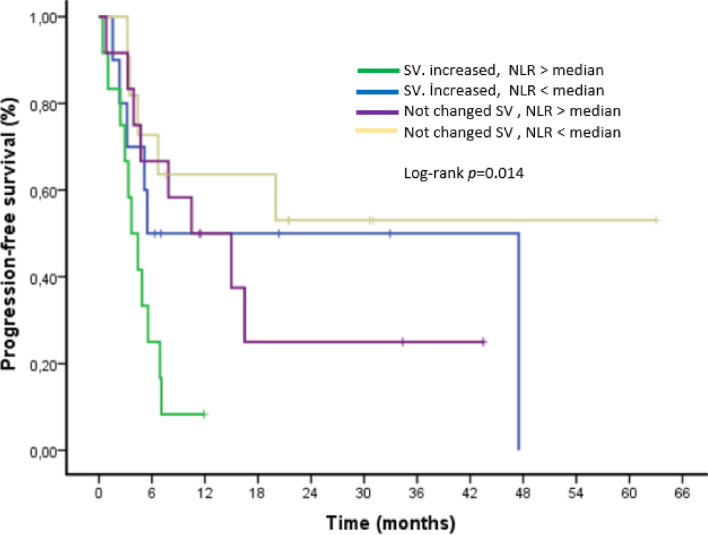
Kaplan–Meier curve for progression-free survival (PFS) stratified on Neutrophil Lymphocyte Ratio( NLR)

**Fig. 5:**
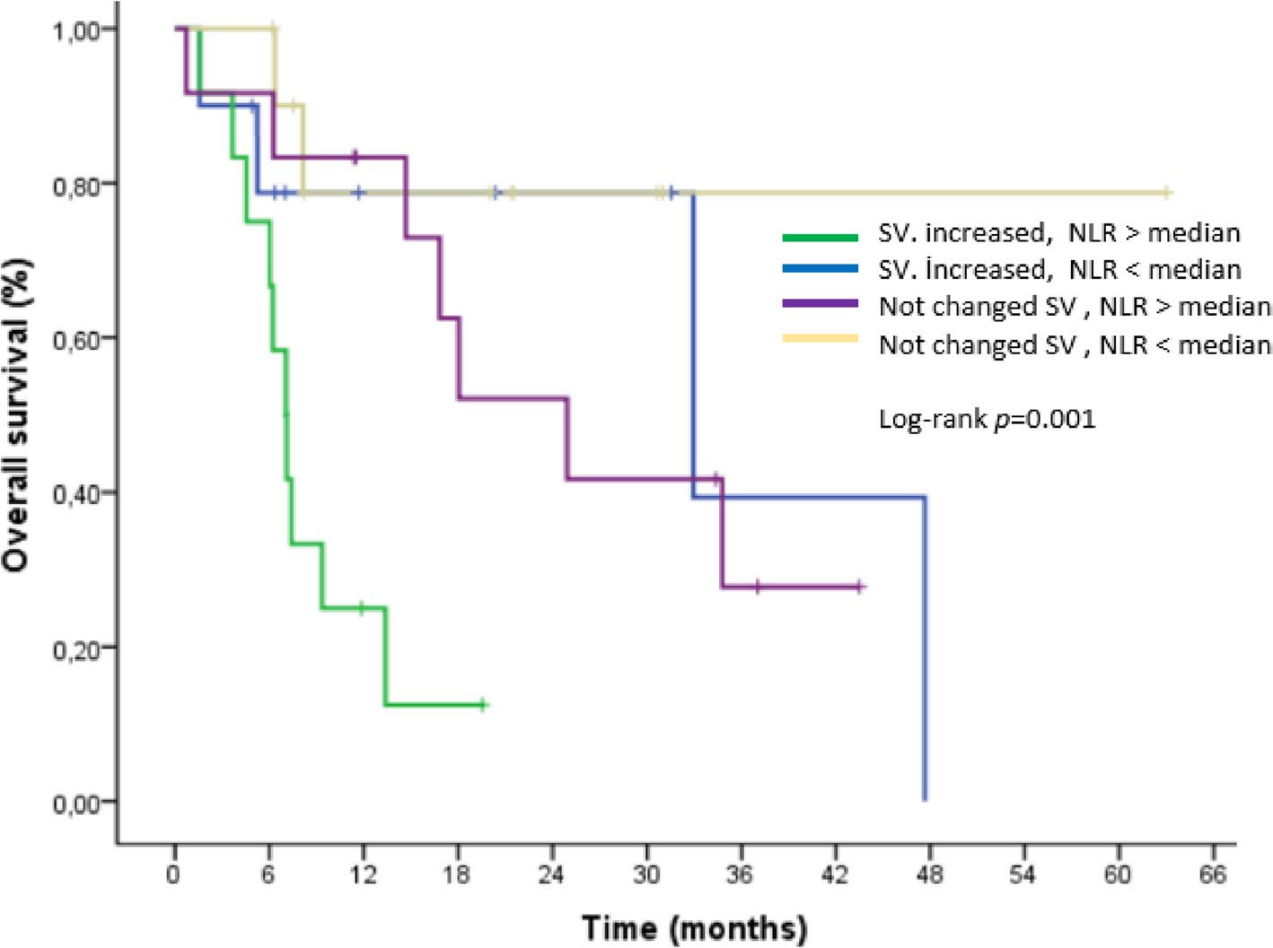
Kaplan–Meier curve for overall survival (OS) stratified on Neutrophil Lymphocyte Ratio( NLR)

Increasing spleen volume was associated with the lowest survival rates and NLR above the median, according to an examination of increased splenic volume across subgroups of NLR (Figs. 4 and 5).

**Fig. 6:**
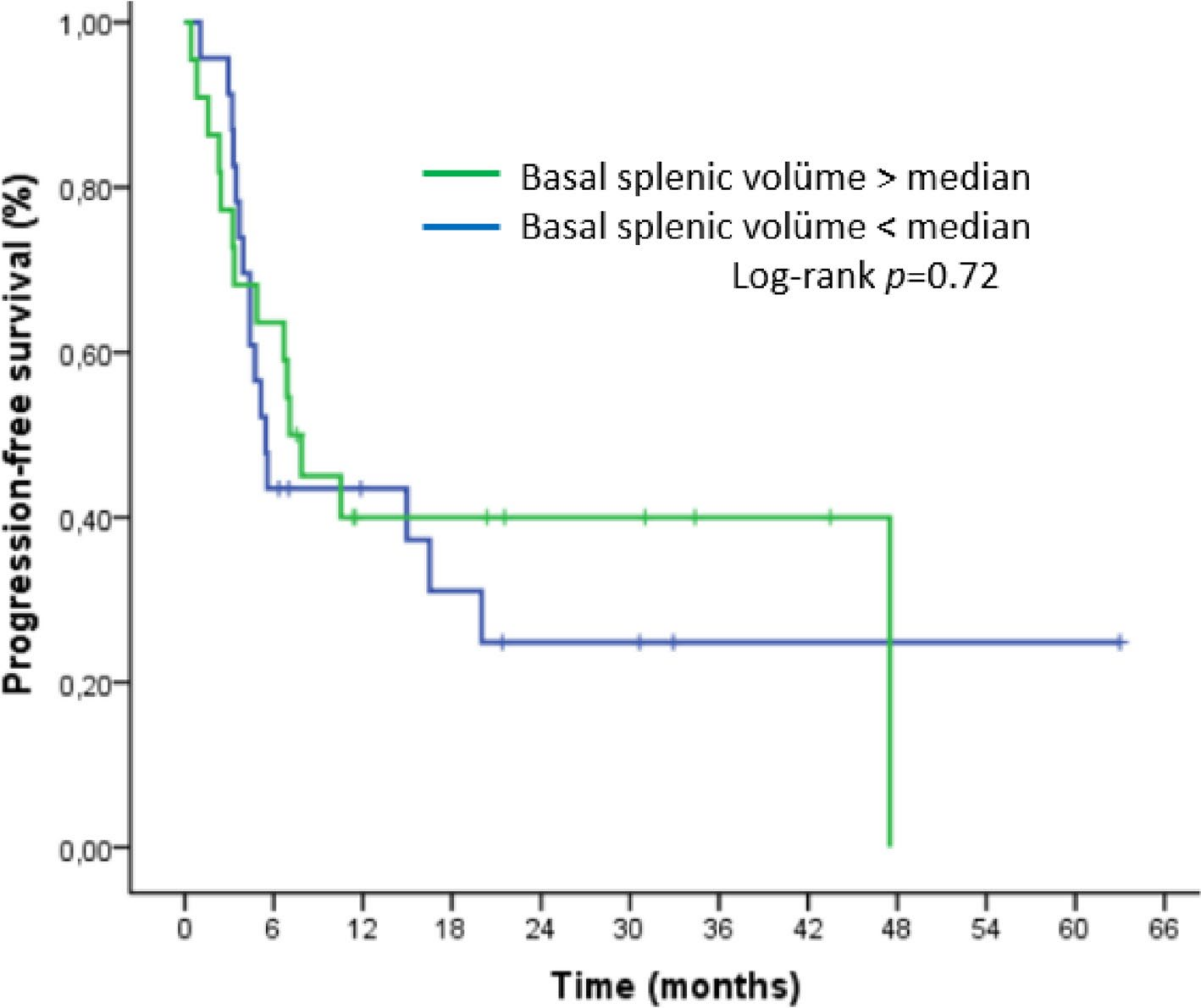
Kaplan–Meier curve for progression-free survival (PFS) according to basal splenic volume at the beginning of nivolumab treatment

**Fig. 7:**
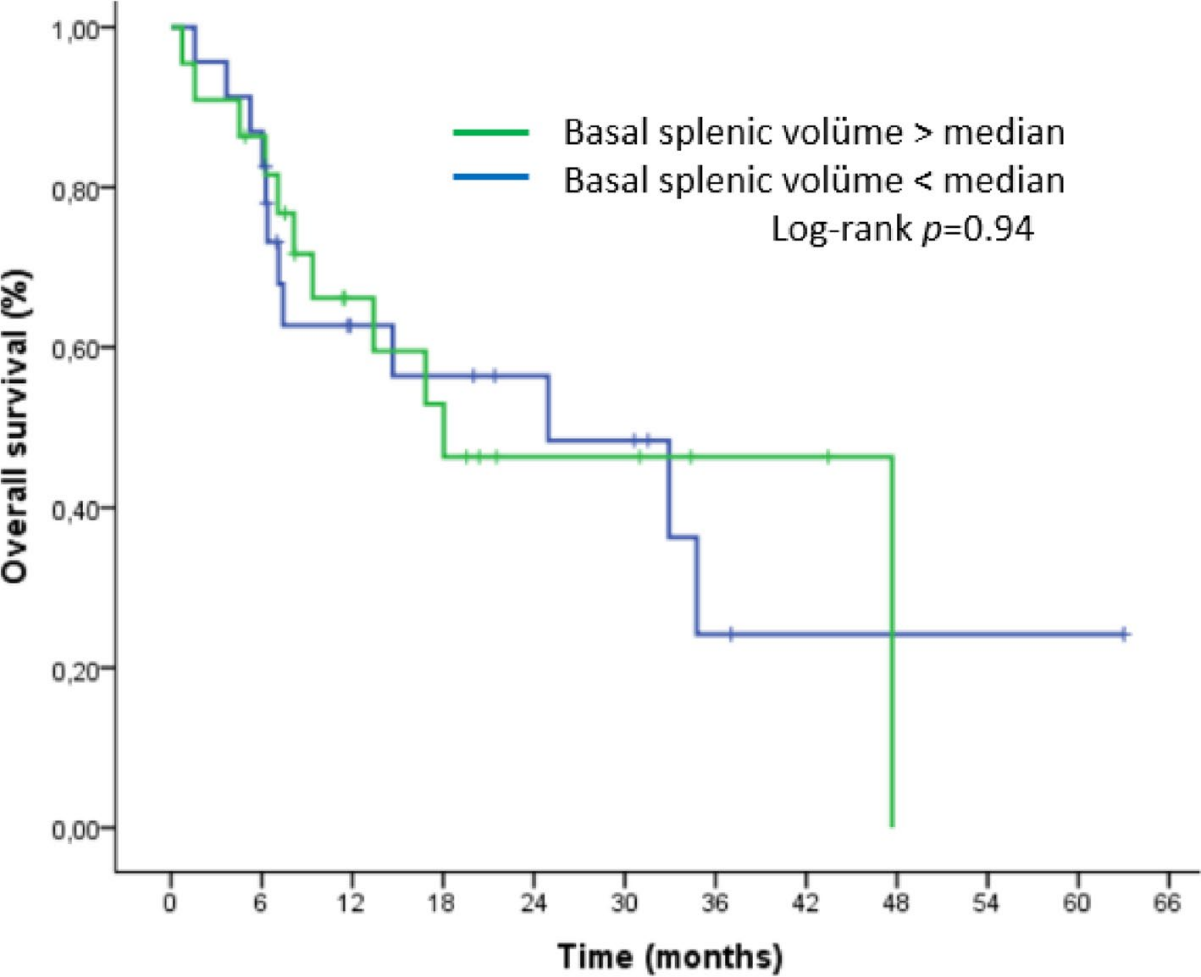
Kaplan–Meier curve for overall survival (OS) according to basal splenic volume at the beginning of nivolumab treatment

Basal splenic volume was analyzed separately. However, neither PFS nor OS showed a significant difference (Figures 6 and 7).

**Fig. 8:**
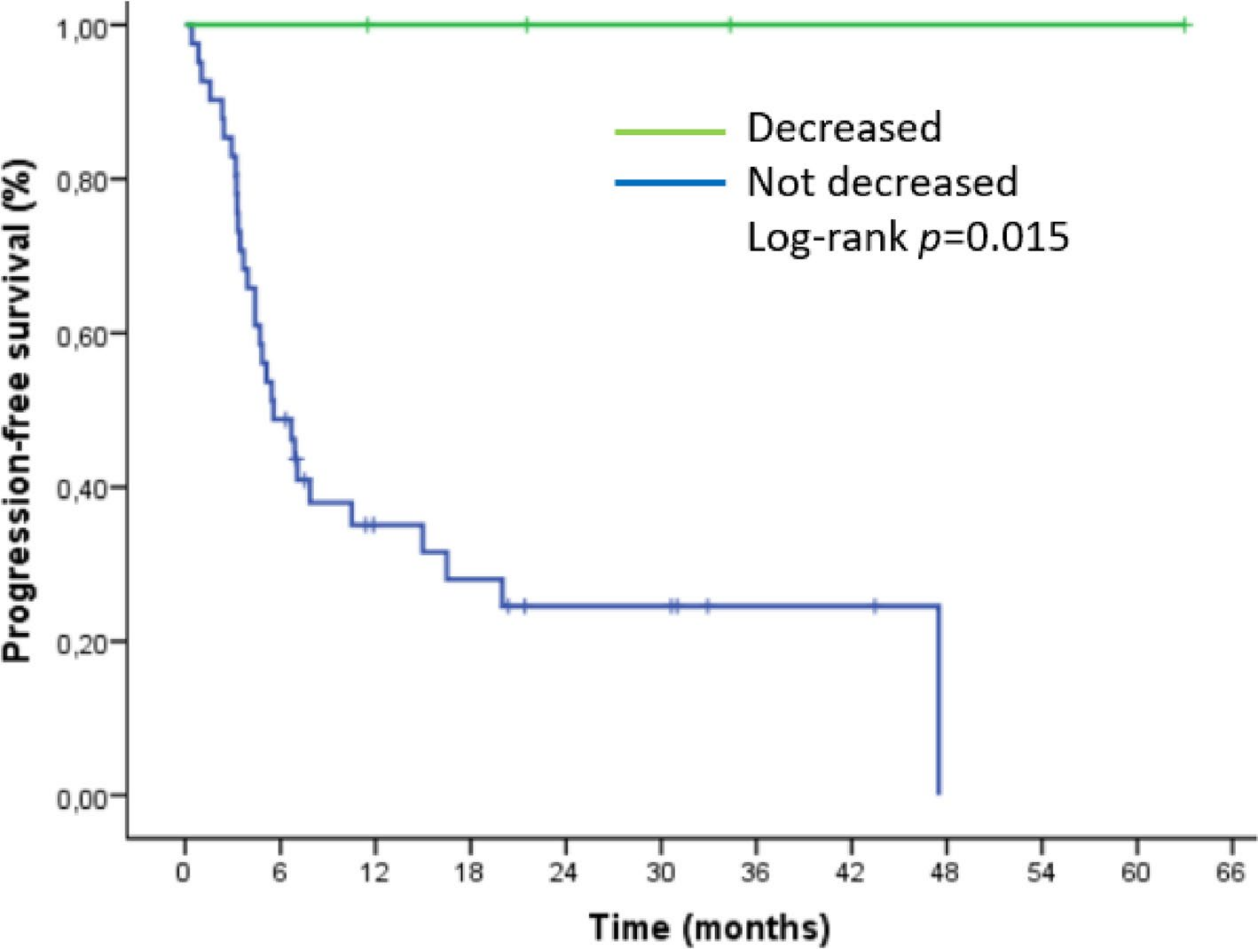
Kaplan-Meier curve for progression-free survival (PFS) based on splenic volume decrease after nivolumab initiation

**Fig. 9:**
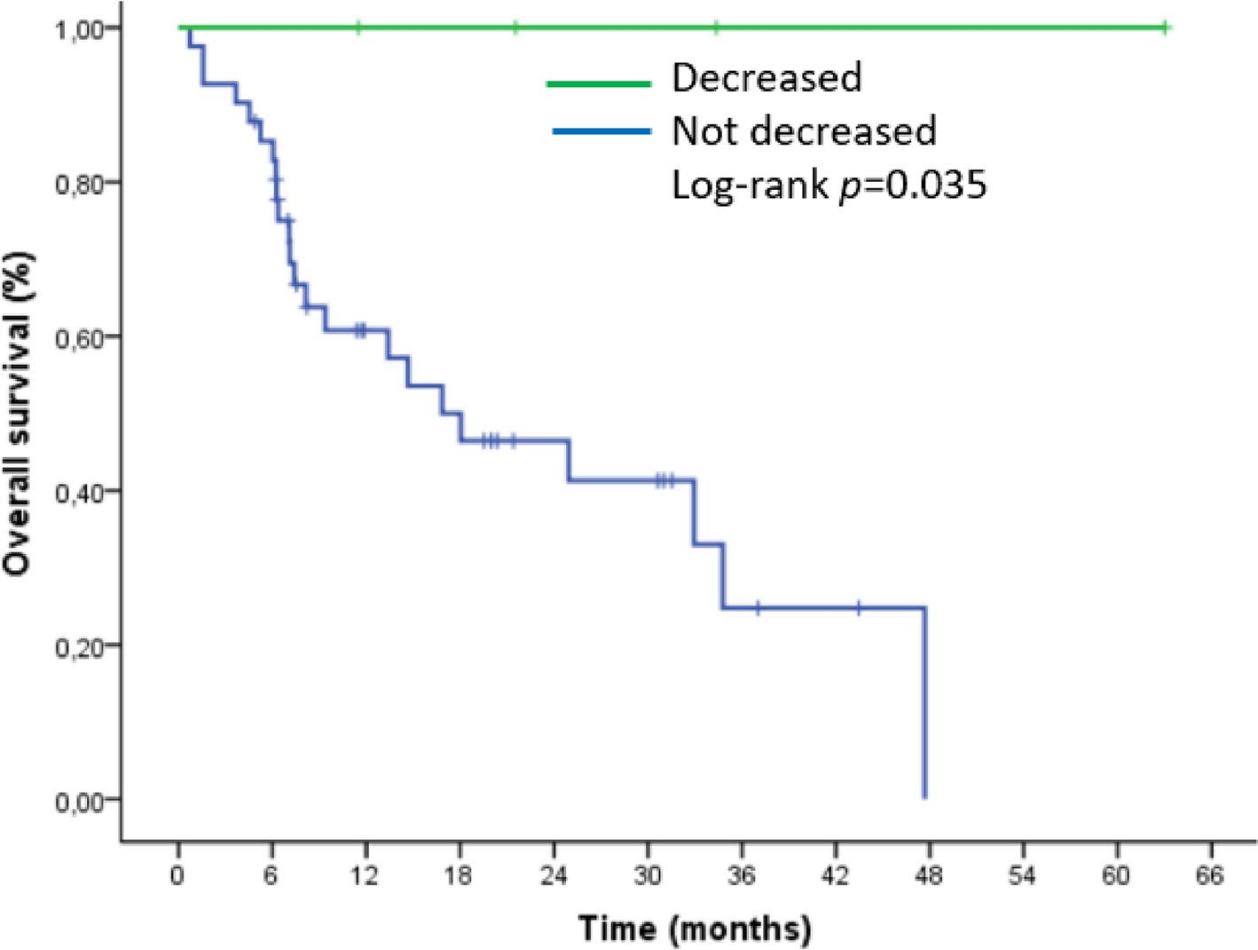
Kaplan–Meier curve for overall survival (OS) based on splenic volume decrease after nivolumab initiation

In four patients with decreased splenic volume, neither PFS nor OS could reach the median value. Log-rank *p* value in respectively (0.015, 0.035) (Figs. 8 and 9).

## Discussion

This study demonstrates that the increase in splenic volume after initiation of nivolumab therapy may be a predictive marker for decreased survial in patients with mRCC who received immunotherapy alone. Previous preclinical and clinical studies have shown that this increase in splenic volume may be an indirect sign of increased MDSC cells. In our study, changes in splenic volume were found to be predictive of immunotherapy response independently of inflammatory parameters such as NLR and PLR, which can explain the development of splenomegaly. In addition, to our knowledge, this is the first study to reveal a relationship between spleen size and survival in metastatic RCC.

There are many studies in the literature showing that increased MDSC cells are associated with immunotherapy resistance and that MDSC-targeted therapies improve immunotherapy responses. The measurement of MDSC is costly, time-consuming, and not yet standardized, but the measurement of splenic volume is quick and simple; thus, we used this hypothesis in our investigation. According to findings of our study increased spleen volume might be associated with an increase in MDSC cells.

Looking at previous studies in different cancer types, Lock Galland et al. reported a change in median splenic volume of 4.4% in patients treated with ICIs for metastatic non-small cell lung cancer (NSCLC). They also showed that this change in splenic volume might serve as a predictor of overall survival. In addition, they found that the basal splenic volume measured prior to the immunotherapy was associated with overall survival (OS). The authors primarily associated the result of the study with chronic inflammation [18]. In our study, the median change in splenic volume was found as 10%, and changes above the median value were considered as significant. The results of this research are similar to ours. However, unlike this study, in the present study differences in basal splenic volume measured before starting nivolumab were not found to be effect on survival. This difference may be related to the treatments that mRCC patients receive until they received immunotherapy.

The preclinical and clinical studies have shown that MDSC cells accumulated in the tumor microenvironment are associated with sunitinib resistance in patients who progress while using sunitinib in RCC patients [12]. Considering our patient population, the reason for the higher baseline splenic volume compared to the normal population can be explained by the effect of increased MDSC cells with previous treatments, because before nivolumab, all of the our patients had been treated with at least one TKI regimen. Therefore, when nivolumab treatment is started in patients with high MDSC rate and increased splenic volume, the altered immune composition and the chronic inflammation accompanying this process will reduce the effectiveness of the newly started immunotherapy and will activate the resistance mechanisms.

However, if the tumor microenvironment may change in favor of the immune system with the effect of newly started ICI treatment, especially in patients without chronic inflammation, as a result MDSCs and the inhibitory pathways they initiate will not increase, and may even decrease in some patients. In particular, the increased survival results in our patient population with decreased splenic volume or with increased splenic volume not accompanied by a high Neutrophil Lymphocyte Ratio (NLR) support this hypothesis and further studies are needed on this subject.

There are also studies that contradict our findings. For example, in a study by Francesca Castagnoli et al. in patients with advanced NSCLC, it was found that changes in splenic volume in patients treated with pembrolizumab had no predictive or prognostic value [19].

Susok et al. [20] studied the effect of treatment on splenic volume in advanced melanoma patients. Compared with baseline measurements, they observed a significant increase in median splenic volume after 3 months in patients treated with ICIs, but they reported that this result was unrelated to clinical parameters.

Again, Lukas Müller at all [21]. found that a large proportion of hepatocellular carcinoma (HCC) patients who received immunotherapy treatment as first-line therapy had an increase in splenic volume after initiation of immunotherapy, but this was not associated with survival outcomes. They reported that this increase in splenic volume was mostly due to portal hypertension, not immune modulation.

We think that the difference of our study from these studies is due to its relationship with chronic inflammation. We would like to emphasize that one of the most important mechanisms of immune resistance that develops against ICI-based therapy initiated on the basis of chronic inflammation is the increase in MDSC cells, and this increase can be easily detected by splenomegaly developing after ICI is started. In addition, NLR is known to be one of the peripheral indicators of chronic inflammation and there is a large literature on this subject [22–24].

In chronic inflammation, MDSCs increase with the effect of some cytokines such as Interleukin-1β (IL-1β). By suppressing the cell activities of T cells and NK cells, elevated MDSCs increase tumor development and contribute to ICI resistance. MDSCs stimulate the production of Treg cells and increase the release of Immunosuppressive cytokines, such as interleukin-10 (IL-10), which specifically suppresses the activities of CD4 + and CD8 + T cells and promotes tumor growth [25]. Consistent with this literature, in our patient population, the group with increased splenic volume accompanied by an increase in NLR was found to be the group with the worst survival results.

There were several limitations of the current research. First, a retrospective design of the study, limited number of patients were included in the study. Due to the retrospective nature of our study, serum samples from that period were unavailable; thus, simultaneous measurements of MDSCs could not be performed alongside splenic volume assessments. Due to the reimbursement issues in our country, our patients were received nivolumab as a second- or third-line therapy. Moreover, our research participants represented the IMDC risk categories heterogeneously, with the majority belonging to the intermediate risk category. The major strength of the current study is that it is the first data analysis to investigate the association between immunotherapy and changes in spleen volume in patients with mRCC.

## Conclusion

In conclusion, our results suggest that the change in splenic volume throughout immunotherapy regimens may be utilized to predict PFS and OS in mRCC patients undergoing treatment. Chronic inflammation and MDSC accumulation, which are known to be associated with immunotherapy resistance, appear to be related to splenic volume. To confirm these findings, prospective immunotherapy clinical trials that concurrently analyze the relationship between changes in splenic volume and MDSC are necessary.

## Data Availability

The datasets used and/or analysed during the current study available from the corresponding author on reasonable request.

## References

[1] Simonaggio A, Simonaggio A, Epaillard N, Pobel C, Moreira M, Oudard S, Vano Y-A (2021). Tumor microenvironment features as predictive biomarkers of response to immune checkpoint inhibitors (ICI) in metastatic clear cell renal cell carcinoma (mccRCC). Cancers..

[2] Gooden MJ (2011). The prognostic influence of tumour-infiltrating lymphocytes in cancer: a systematic review with meta-analysis. Br J Cancer.

[3] Tucker MD, Rini BI (2020). Predicting response to immunotherapy in metastatic renal cell carcinoma. Cancers..

[4] Gabrilovich DI (2017). Myeloid-derived suppressor cells. Cancer Immunol Res.

[5] Liu C, Liu C, Liu R, Wang B, Lian J, Yao Y, Sun H, Zhang C, Fang L, Guan X, Shi J, Han S, Zhan F, Luo S, Yao Y, Zheng T, Zhang Y (2021). Blocking IL-17A enhances tumor response to anti-PD-1 immunotherapy in microsatellite stable colorectal cancer. J Immunother Cancer.

[6] Kuebler H (2006). Immature myeloid cell (ImC)-mediated immunosuppression in advanced renal cell cancer (RCC). J Clin Oncol.

[7] Pastaki Khoshbin A, Pastaki Khoshbin A, Eskian M, Keshavarz-Fathi M, Rezaei N (2019). Roles of myeloid-derived suppressor cells in cancer metastasis: immunosuppression and beyond. Arch Immunol Ther Exp.

[8] Veglia F, Sanseviero E, Gabrilovich DI (2021). Myeloid-derived suppressor cells in the era of increasing myeloid cell diversity. Nat Rev Immunol.

[9] Gide TN (2018). Primary and acquired resistance to immune checkpoint inhibitors in metastatic melanoma resistance to immunotherapy in melanoma. Clin Cancer Res.

[10] Highfill SL, et al. Disruption of CXCR1-mediated MDSC Tumor trafficking enhances anti-PD1 efficacy. Sci Transl Med. 2014;6(237):237ra67. 10.1126/scitranslmed.3007974 PMC6980372 24848257

[11] Taylor NA, Taylor NA, Vick SC, Iglesia MD, Brickey WJ, Midkiff BR, McKinnon KP, Reisdorf S, Anders CK, Carey LA, Parker JS, Perou CM, Vincent BG, Serody JS (2017). Treg depletion potentiates checkpoint inhibition in claudin-low breast cancer. J Clin Investig.

[12] Finke J, Finke J, Ko J, Rini B, Rayman P, Ireland J, Cohen P (2011). MDSC as a mechanism of tumor escape from sunitinib mediated anti-angiogenic therapy. Int Immunopharmacol.

[13] Haka AS, Haka AS, Volynskaya Z, Gardecki JA, Nazemi J, Lyons J, Hicks D, Fitzmaurice M, Dasari RR, Crowe JP, Feld MS (2006). In vivo margin assessment during partial mastectomy breast surgery using Raman spectroscopy. Cancer Res.

[14] Bronte V, Pittet MJ (2013). The spleen in local and systemic regulation of immunity. Immunity.

[15] Ramudo L (2009). Signal transduction of MCP-1 expression induced by pancreatitis‐associated ascitic fluid in pancreatic acinar cells. J Cell Mol Med.

[16] Limagne E (2016). Accumulation of MDSC and Th17 cells in patients with metastatic colorectal cancer predicts the efficacy of a FOLFOX–Bevacizumab drug treatment RegimenMDSC and Th17 in FOLFOX–Bevacizumab–treated mCRC patients. Cancer Res.

[17] Prassopoulos P, Prassopoulos P, Daskalogiannaki M, Raissaki M, Hatjidakis A, Gourtsoyiannis N (1997). Determination of normal splenic volume on computed tomography in relation to age, gender and body habitus. Eur Radiol.

[18] Galland L, Galland L, Lecuelle J, Favier L, Fraisse C, Lagrange A, Kaderbhai C, Truntzer C, Ghiringhelli F (2021). Splenic volume as a surrogate marker of Immune checkpoint inhibitor efficacy in metastatic non small cell lung cancer. Cancers.

[19] Castagnoli F, Castagnoli F, Doran S, Lunn J, Minchom A, O’Brien M, Popat S, Messiou C, Koh D-M (2022). Splenic volume as a predictor of treatment response in patients with non-small cell lung cancer receiving immunotherapy. PLoS ONE.

[20] Susok L, Susok L, Reinert D, Lukas C, Stockfleth E, Gambichler T (2021). Volume increase of spleen in melanoma patients undergoing immune checkpoint blockade. Immunotherapy.

[21] Müller L, Müller L, Gairing SJ, Kloeckner R, Foerster F, Weinmann A, Mittler J, Stoehr F, Emrich T, Düber C, Galle PR, Hahn F (2022). Baseline splenic volume outweighs Immuno-modulated size changes with regard to survival outcome in patients with hepatocellular carcinoma under Immunotherapy. Cancers.

[22] Balkwill FR, Mantovani A. Cancer-related inflammation: common themes and therapeutic opportunities. Semin Cancer Biol. 2012;22(1):33–40. Academic Press, Elsevier. 10.1016/j.semcancer.2011.12.005 22210179

[23] Boissier R, et al. The prognostic value of the neutrophil-lymphocyte ratio in renal oncology: a review. Urol Oncol. 2017. Elsevier. 10.1016/j.urolonc.2017.01.016 28233671

[24] Bilen MA, Bilen MA, Martini DJ, Liu Y, Lewis C, Collins HH, Shabto JM, Akce M, Kissick HT, Carthon BC, Shaib WL, Alese OB, Pillai RN, Steuer CE, Wu CS, Lawson DH, Kudchadkar RR, El‐Rayes BF, Master VA, Ramalingam SS, Owonikoko TK, Harvey RD (2019). The prognostic and predictive impact of inflammatory biomarkers in patients who have advanced-stage cancer treated with immunotherapy. Cancer.

[25] Law AM, Valdes-Mora F, Gallego-Ortega D (2020). Myeloid-derived suppressor cells as a therapeutic target for cancer. Cells.

